# A scoping review on the epidemiology and public significance of *Brucella abortus* in Chinese dairy cattle and humans

**DOI:** 10.1016/j.onehlt.2024.100683

**Published:** 2024-01-26

**Authors:** Yu Wang, Emilie Vallée, Cord Heuer, Youming Wang, Aizhen Guo, Zhen Zhang, Chris Compton

**Affiliations:** a EpiCentre, School of Veterinary Science, Massey University, Private Bag 11-222, Palmerston North, New Zealand; b China Animal Health and Epidemiology Center, Qingdao, China; c National Key Laboratory of Agricultural Microbiology, Hubei Hongshan Laboratory, College of Veterinary Medicine, Huazhong Agricultural University, Wuhan 430070, China; d Henan Dairy Herd Improvement Center, Zhengzhou, Henan 450045, China

**Keywords:** Dairy cattle, *Brucella abortus*, Diagnosis, Epidemiology, One health, Control strategy

## Abstract

Brucellosis, caused by *Brucella* spp., is a re-emerging One Health disease with increased prevalence and incidence in Chinese dairy cattle and humans, severely affecting animal productivity and public health. In dairy cattle, *B. abortus* is the primary causative agent although infections with other *Brucella* species occur occasionally. However, the epidemiological and comparative importance of *B. abortus* in dairy cattle and humans remains inadequately understood throughout China due to the heterogeneity in locations, quality, and study methods. This scoping review aims to describe the changing status of *B. abortus* infection in dairy cattle and humans, investigate the circulating *Brucella* species and biovars, and identify factors driving the disease transmission by retrieving publicly accessible literature from four databases. After passing the prespecified inclusion criteria, 60 original articles were included in the final synthesis. Although the reported animal-level and farm-level prevalence of brucellosis in dairy cattle was lower compared to other endemic countries (e.g. Iran and India), it has been reported to increase over the last decade. The incidence of brucellosis in humans displayed seasonal increases. The Rose Bengal Test and Serum Agglutination Test, interpreted in series, were the most used serological test to diagnose *Brucella* spp. in dairy cattle and humans. *B. abortus* biovar 3 was the predominant species (81.9%) and biovar (70.3%) in dairy cattle, and *B. melitensis* biovar 3 was identified as the most commonly detected strain in human brucellosis cases. These strains were mainly clustered in Inner Mongolia and Shannxi Province (75.7%), limiting the generalizability of the results to other provinces. Live cattle movement or trade was identified as the key factor driving brucellosis transmission, but its transmission pattern remains unknown within the Chinese dairy sector. These knowledge gaps require a more effective One Health approach to be bridged. A coordinated and evidence-based research program is essential to inform regional or national control strategies that are both feasible and economical in the Chinese context.

## Highlights

• A comprehensive scoping review examined the seroprevalence of brucellosis in dairy cattle and humans.
• *B. abortus* biovar 3 occupied major brucellosis in dairy cattle, while *B. melitensis* biovar 3 dominated in humans in China.
• The review identified heterogeneous research quality and knowledge gaps to refine the future research in China.
• A “One Health” approach is required to guide effective control schemes for *B. abortus* in dairy cattle in China.

## 1 Introduction

Brucellosis, mainly caused by *Brucella* species *(Brucella* spp.*)*, is globally recognized as a significant zoonotic and One Health disease [1]. *Brucella* spp. contains more than twelve species of intracellular bacteria; among them four zoonotic species infect multiple hosts, such as cattle and small ruminants [2]. *Brucella* spp. exhibits host tropism but is not restricted to an exclusive host; *B. abortus* mainly infects cattle, whereas *B. melitensis* primarily affects small ruminants [2]. Characteristic clinical signs of *B. abortus* infection in cattle and small ruminants are abortion, retained placenta, orchitis, infertility, and reduced milk yield [1,3,4]. In humans, the symptoms of this disease include muscle pain, arthritis, rising and falling “undulant” fever, hyperhidrosis, fatigue, and night sweats [5]. Given the profound impact of *B. abortus* on humans and animals, the World Health Organisation (WHO) and World Organisation for Animal Health (WOAH, founded as OIE) have classified *B. abortus* as a notifiable or listed disease affecting the international trade in animals and their products [6,7].

*B. abortus* is a highly contagious pathogen that can spread across multiple hosts. The primary transmission routes of *B. abortus* for cattle are by direct contact with aborted products or vaginal secretions of infected animals or consumption of unpasteurized milk [8,9]. Contacting wild animals (e.g., rodents and deer) and ticks may also provoke the re-emergence of *B. abortus* infection in domestic animals [10,11]. Direct contact with infected animals and their aborted products without wearing personal protection equipment (PPE, e.g., gloves and masks) is considered the riskiest way to acquire brucellosis in humans [12,13]. Consumption of raw or unpasteurized dairy products and inhaling *Brucella* aerosols are common routes of *Brucella* spp. acquisition in humans [1,[14], [15], [16]]. Therefore, *B. abortus* is regarded as a One Health hazard to animals, occupation-related workers, and the public.

Since almost all cases of brucellosis in humans are of animal origin, prevention of brucellosis in humans could be achieved through either human hygiene measures or control measures in infected animal populations. Controlling the spread of *B. abortus* on farms is often the most efficient and cost-effective approach to mitigate public health and food safety risks compared to traditional hygiene measures at the processing stage [[17], [18], [19]]. Intensified surveillance in animal host species, test-and-slaughter, restriction of live animal movement, and mass vaccination are effective brucellosis control strategies [20]. Australia and New Zealand have successfully eradicated *B. abortus* from domestic farms by implementing extensive mandatory vaccination, followed by a rigorous test-and-culling approach [21,22]. In areas where *B. abortus* is endemic, vaccinating susceptible animals is highly recommended by WOAH to provide protection against virulent *Brucella* spp. strains and reduce disease impacts on reproduction and production [6].

*B. abortus* is still endemic in many countries, with a particularly high prevalence in Africa and Asia, including China [23,24]. Given China's substantial population of 3.09 million dairy cows and 11 million livestock workers, addressing this production-limiting and zoonotic disease at the population level is crucial for economic, food security, and One Health [25]. However, *B. melitensis* is widely considered as primary concern in humans [26,27], indirectly resulting in *B. abortus* being overlooked in both dairy cows and humans for a long time. There is still controversy over the the relative importance of *B. abortus* to *B. melitensis* in cows and humans and whether to adopt more intense control measures in cows in China. A comprehensive understanding of the current epidemiology of *B. abortus* in dairy cattle and humans is a prerequisite for making evidence-based decisions about this pathogen and facilitating the comparative opportunities in facilitating brucellosis One Health management. However, existing literature reports are heterogeneous in geographical location, study design, and reporting quality, making it difficult to assess the overall *B. abortus* status in China. A scoping review is a type of literature review that transparently and reproducibly integrates the current literature on a related topic with little or no research done and can also be a precursor to a subsequent systematic review [28]. Although many works of literature report brucellosis in China, there has not been a formal scoping review on *B. abortus* in dairy cows and humans. This scoping review aimed to: (1) provide an overview of *B. abortus* status in Chinese dairy cattle and human populations between January 2004 and December 2022; and (2) identify the key factors driving the spread of *B. abortus*. The insights gleaned from this review can be used to tailor evidence-based control strategies to reduce the disease burden both in dairy farms and humans in China.

## 2 Methods

### 2.1 Literature search strategy

**Fig. 1:**
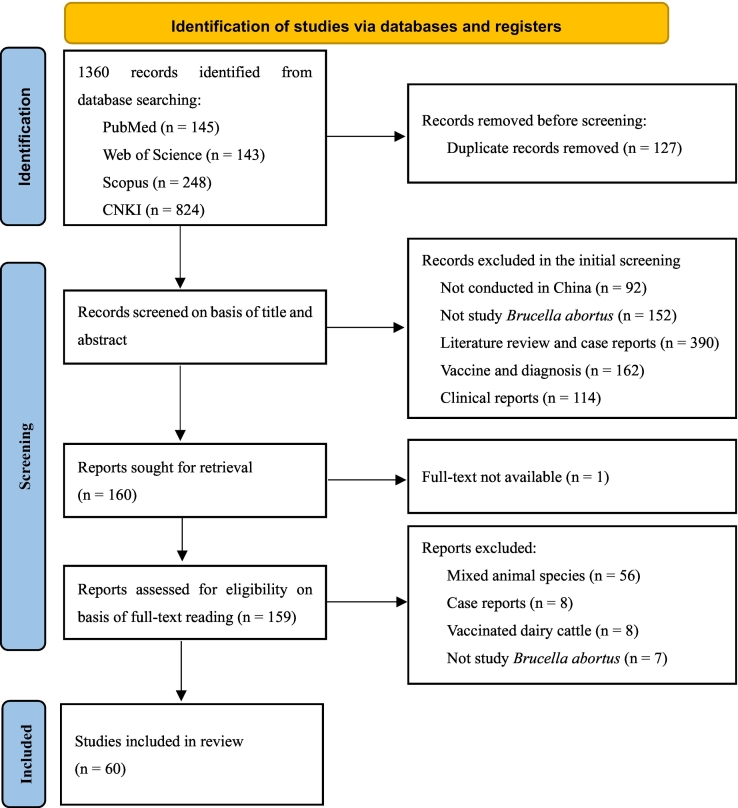
PRISMA flow diagram of the studies identified, screened, assessed, and included in a scoping review of brucellosis in dairy cattle and humans in China.

Following the Preferred Reporting Items for Systematic reviews and Meta-Analyses (PRISMA) guidelines [29], a comprehensive literature search was conducted to identify scientific articles published from 1st January 2004 to 31st December 2022 using four databases (PubMed, Web of Science, Scopus, China National Knowledge Infrastructure - CNKI) as shown in Fig. 1. A complete list of the search terms and their combinations used for each database is available in Supplemental Table S1. The reference management software program EndNote X9 (Clarivate Analytics, Philadelphia, PA) was used to organize and remove duplicate publications retrieved from the four databases.

### 2.2 Study inclusion and exclusion criteria

The inclusion and exclusion criteria for literature retrieval were detailed in Table 1. Studies that reported the prevalence of antibodies against *B. abortus* in dairy cows in China with explicit diagnostic tests were included. Reviews and clinical reports that only described the clinical symptoms and treatment were excluded because they were not relevant to the scope of this review. An initial screening for inclusion was made according to the titles and abstracts, and publications that reported on other countries, species, and diseases were removed immediately. The full-text manuscripts were then read before the final decision was made to include or exclude them in this review according to the criteria in Table 1. Details of the specific reasons for exclusion during the two-step screening process can be seen in Fig. 1. YW initially conducted the literature selection and data extraction and discussed with other authors to reach a consensus if there were any uncertainties. However, given the language barriers for some authors regarding literature written in Chinese, the main work for retrieved Chinese literature was completed by YW.

**Table 1:** Inclusion and exclusion criteria used for literature screening in this study.

Inclusion	Exclusion
Study conducted in China	Study conducted elsewhere
Study dairy cows or humans	Study other hosts
Clearly defined study year and province	Unclear study time and place
Tested for *Brucella abortus* using explicit tests	Study not investigating *Brucella abortus*
Original observational studies	Review and clinical reports
Full text available	Full text unavailable

### 2.3 Data extraction

A template was created to record information about the methodology and results of each publication. The data set documented general study characteristics, including author, publication year, investigation time, province, diagnostic tests used, criteria for positivity, number of units and group (i.e., animals and farms), animal-level and farm-level prevalence, and study type. Sampling times, province, species and biovars of strains, and identification methods were recorded for studies that reported molecular characterization. Only confirmed isolated strains were counted with relevant species and biovars identification methods. Sample size, study population, study type, and statistical methods used were documented for risk factor analysis studies. Data on the monthly number of notified human cases and annual incidence rates from January 2004 to December 2022 were retrieved from the Data Center of China Public Health Science (https://www.phsciencedata.cn/Share/en/index.jsp). Human brucellosis diagnosis and notification protocols have been described elsewhere [13,27].

## 3 Results

### 3.1 Characteristics of included studies

The initial literature searching identified 1360 records from four databases, of which 127 were removed for duplication. After screening the titles and abstracts, 1073 of them were rejected for one or more reasons, including mixing dairy cows with other species, ambiguous study places or times, not declaring diagnostic tests used, and others, as detailed in Fig. 1. Literature reviews and case reports (390/1073) accounted for about a third of the reasons for exclusion. Full-text assessment of the articles was made on 159, and 60 studies were finally enrolled for data extraction (Fig. 1). Articles written in either Chinese or English were retrieved, of which forty-four (44/60) were in Chinese.

### 3.2 Brucellosis prevalence estimation studies in dairy cattle

**Fig. 2:**
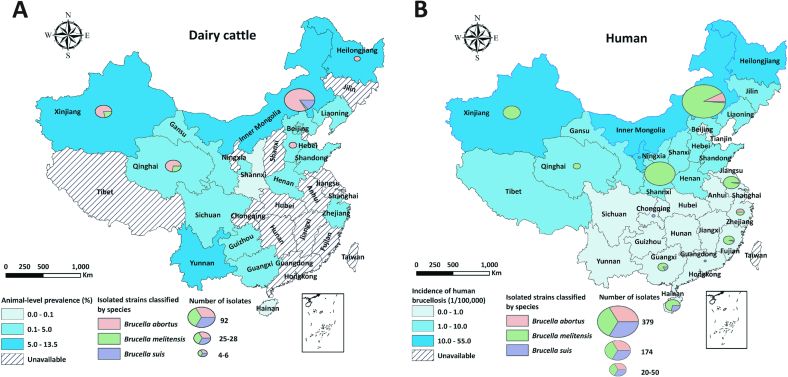
Geographical distribution of reported animal-level prevalence of brucellosis in dairy cattle (Panel A) and incidence of human brucellosis in 2019 (Panel B) with respective count of strain isolation and species identification, in China from 60 studies identified from a scoping review.

Thirty-two reports investigated the prevalence of antibodies against *B. abortus* in dairy cattle. Table 2 summarizes the main characteristics of these studies. Major studies were conducted in a single province, covering 17 of 32 provinces or autonomous regions in China (Fig. 2). The cross-sectional design was employed in 72% of the studies, followed by cohort studies. A combined Rose Bengal Test (RBT) and Serum Agglutination Test (SAT) interpreted in series was used in 84% of the studies (27/32) to judge *B. abortus* status. About half of the studies investigated mixed-type (comprising both large-scale and smallholder) dairy farms, while 30 % of studies did not report herd size. Sampling frame and method were unavailable in 47% of the studies (15/32), and census (6/32) and stratified random sampling (6/32) were the usual methods in the remaining studies. The age of sampled animals was not reported in two-thirds of the studies (21/32), and other studies sampled groups with varying minimum ages, ranging from 3 months to >2 years of age. The median of all reported animal-level prevalences was 2.1% (range: 0.0% – 13.5%), while at the farm level its median prevalence increased to 10.2% (range: 0.0%–100%). However, only half of the studies reported farm-level prevalence of brucellosis.

**Table 2:** Characteristics of retrieved studies that investigated the seroprevalence of brucellosis in Chinese dairy cows, including study design, geographical location, diagnostic tests, population selection and age, and sample size.

Study period	Study type	Province	Sample type	Diagnostic tests	Herd size	Population selection	Age group	Sample size^⁎^	Animal-level prevalence (%)	Herd-level prevalence (%)	References
2000–2009	Cohort	Beijing	Blood	RBT + SAT	Mixed	Census	Census	173,024	0.1	–	[30]
2003–2013	Cohort	Guangxi	Blood	RBT + SAT	Not stated	Not stated	Not stated	1039	2.7	–	[31]
2004–2010	Cohort	Zhejiang	Blood	RBT + SAT	Mixed	Census	Census	110,220	1.5	–	[32]
2007	Cross-sectional	Zhejiang	Blood	RBT + SAT	Not stated	Not stated	Not stated	1071	2.4	–	[33]
2007–2011	Cohort	Qinghai	Blood	RBT + SAT	Not stated	Not stated	Not stated	138,350	0.3	–	[34]
2009–2011	Cross-sectional	15 provinces	Milk	PCR	Large-scale	Stratified randomized	> 2 years	5211	1.1	–	[35]
2009–2011	Cross-sectional	Inner Mongolia	Blood	RBT + SAT	Mixed	Not stated	Not stated	5875 (196)	6.2	26.5	[36]
2009–2013	Cohort	Gansu	Blood	RBT + SAT	Not stated	Not stated	Not stated	10,742	2.0	–	[37]
2012	Cross-sectional	Shandong	Blood	RBT + SAT	Mixed	Census	> 3 months	30,119 (1803)	0.4	2.6	[38]
2012	Cross-sectional	Shandong	Blood	RBT + SAT	Not stated	Not stated	Not stated	333	1.5	–	[39]
2012	Cross-sectional	Qinghai	Blood	RBT + SAT	Not stated	Stratified randomized	Not stated	18,282	0.2	–	[40]
2012–2013	Cross-sectional	Shandong	Blood	RBT + SAT	Mixed	Simple randomized	Not stated	485 (13)	4.9	15.4	[41]
2012–2013	Cross-sectional	Heilongjiang	Blood	RBT + SAT	Mixed	Not stated	Not stated	1590	1.1	–	[42]
2013	Cross-sectional	Liaoning	Blood	RBT + SAT	Mixed	Census	Census	37,888 (187)	0.2	10.2	[43]
2013	Cross-sectional	Hebei	Blood	RBT + SAT	Large-scale	Not stated	> 6 months	4279 (109)	0.1	5.5	[44]
2013–2014	Cross-sectional	Heilongjiang	Blood	I-ELISA + C-ELISA	Large-scale	Not stated	Not stated	4131 (22)	12.3	77.3	[45]
2013–2017	Cohort	Sichuan	Blood	RBT + SAT	Not stated	Stratified randomized	> 12 month	5598	0.2	–	[46]
2013–2017	Cohort	Gansu	Blood	RBT + SAT	Not stated	Not stated	> 8 months	1431 (160)	4.5	7.5	[47]
2014	Cross-sectional	Xinjiang	Blood	RBT + SAT	Large-scale	Not stated	> 6 months	987 (3)	6.5	100.0	[48]
2015	Cross-sectional	Henan	Blood	RBT	Mixed	Census	Census	218 (5)	7.8	100.0	[49]
2015	Cross-sectional	Yunan	Blood	RBT + SAT	Large-scale	Not stated	Not stated	223	13.5	–	[50]
2015–2017	Cohort	Sichuan	Blood	RBT + SAT	Mixed	Census	Census	159,071 (1157)	0.7	19.5	[51]
2015–2017	Cross-sectional	Guizhou	Blood	RBT + SAT	Mixed	Simple randomized	Not stated	25,910 (148)	0.1	4.1	[52]
2017–2018	Cross-sectional	Xinjiang	Blood	RBT + C-ELISA	Smallholder	Stratified randomized	> 2 years	1406	6.8	–	[53]
2017–2019	Cross-sectional	Inner Mongolia	Blood	RBT + SAT	Mixed	Stratified randomized	Not stated	1758	2.7	–	[54]
2018	Cross-sectional	Xinjiang	Blood	RBT + SAT	Mixed	Simple randomized	Not stated	1203	4.7	–	[55]
2018	Cross-sectional	Henan	Blood	RBT + SAT	Mixed	Simple randomized	Not stated	25,088 (581)	0.8	7.2	[56]
2018	Cross-sectional	Shannxi	Blood	RBT + SAT	Not stated	Stratified randomized	Not stated	92 (3)	0.0	0.0	[57]
2019	Cross-sectional	Henan	Blood	RBT + SAT	Mixed	Census	Census	12,755 (68)	2.2	33.8	[58]
2020	Cross-sectional	Hainan	Blood	RBT + SAT	Large-scale	Simple randomized	Census	1690 (2)	0.0	0.0	[59]
2021	Cross-sectional	Shandong	Blood	RBT + SAT	Mixed	Not stated	Not stated	49,080 (1079)	0.5	2.9	[60]
2021	Cross-sectional	Xinjiang	Blood	RBT + SAT	Not stated	Not stated	Not stated	7102	0.6	–	[61]

^⁎^: Number of animals tested (Number of farms tested); −: not available; RBT: Rose Bengal Test; SAT: Serum Agglutination Test; C-ELISA: competitive Enzyme-linked Immunosorbent Assay.

### 3.3 Circulating *Brucella* spp. strains in dairy cattle and humans

**Fig. 3:**
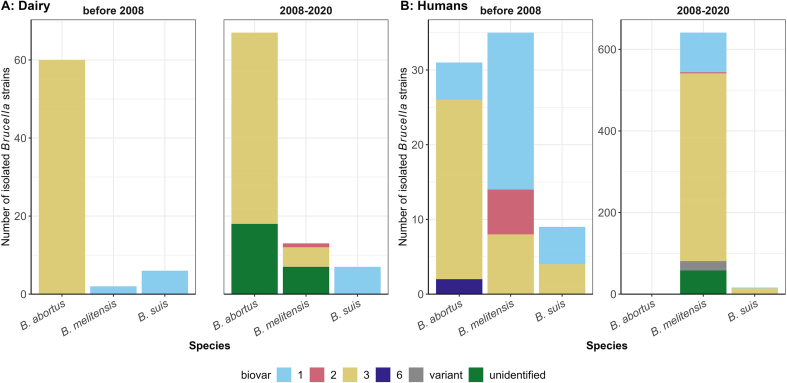
Species and biovars of *Brucella* spp. strains isolated from dairy cattle (Panel A) and humans (Panel B) during 1984–2020, bv represents biovar.

A total of 888 *Brucella* spp. strains were isolated from dairy cattle and humans in 14 provinces of China during 2004–2022 (Table 3, Fig. 2). 733 strains were from humans (across 15 studies) and 155 from dairy cattle (across 8 studies). More *Brucella* strains were isolated after 2008 than before 2008 (745 vs 143, Fig. 3), aligning with the number of studies conducted during each period (16 studies vs 5 studies). Nearly half of the molecular studies (9/21) were conducted in Inner Mongolia, where about half of the *Brucella* isolates (441/888) were acquired. Notably, one study isolated 174 *Brucella* strains in Shannxi Province accounting for 19.6% of the total [62]. *B. melitensis* represented about three-quarters of the total isolates from both humans and dairy cattle, followed by *B. abortus* (18.8%). Specifically, 84.3% of *B. abortus* strains were identified as *B. abortus* biovar 3, while 11.3% were not subjected to specific biovar identification. Over 60% of these *B. abortus* isolates (77/127) were from Inner Mongolia. The proportion of brucellosis attributable to *B. melitensis* in dairy cattle increased markedly from 2.9% before 2008 to 14.9% in 2008–2020 (Fig. 3 Panel A). In humans, *B. melitensis* accounted for over 90% of all *Brucella* isolates, followed by *B. abortus* (4.6%). Before 2008, *B. abortus* and *B. melitensis* were the main etiologies of human brucellosis, and *B. melitensis* biovar 3 stood out after 2008 (Fig. 3 Panel B).

**Table 3:** Characteristics of 846 *Brucella* strains isolated from cattle and humans in China.

Variables	Host		
	Human (%)	Dairy cattle (%)	Total (%)
Total	733 (82.5)	155 (17.5)	888

Species			
*B. abortus*	32 (4.4)	127 (81.9)	159 (17.9)
*B. melitensis*	676 (92.2)	15 (9.7)	691 (77.8)
*B. suis*	25 (3.4)	13 (8.4)	38 (4.3)

*B. abortus* biovars			
Biovar 1	5 (15.6)	0 (0.0)	5 (3.1)
Biovar 3	25 (78.1)	109 (85.8)	134 (84.3)
Biovar 6	2 (6.3)	0 (0.0)	2 (1.3)
Unidentified	0 (0.0)	18 (14.2)	18 (11.3)

*B. melitensis* biovars			
Biovar 1	118 (17.5)	2 (13.3)	120 (17.4)
Biovar 2	9 (1.3)	1 (6.7)	10 (1.4)
Biovar 3	468 (69.2)	5 (33.3)	473 (68.5)
Variant	23 (3.4)	7 (46.7)	30 (4.3)
Unidentified	58 (8.6)	0 (0.0)	58 (8.4)

*B. suis*			
Biovar 1	7 (28.0)	13 (100.0)	20 (52.6)
Biovar 3	17 (68.0)	0 (0.0)	17 (44.7)
Unidentified	1 (4.0)	0 (0.0)	1 (2.6)

Region			
Inner Mongolia	349 (47.6)	92 (59.4)	441 (49.7)
Shannxi	174 (23.7)	0 (0.0)	174 (19.6)
Other provinces	168 (28.6)	63 (40.6)	231 (30.7)

### 3.4 Risk factors for *Brucella abortus* in dairy cattle

Five studies have identified several risk factors associated with *B. abortus* infection in dairy cattle, including a history of abortion, the purchase of new animals, commingling with small ruminants, presence of dogs within a herd, and small herd size [35,38,43,58,63]. The lack of exclusive animal transport vehicles and workers entering sheds without wearing specific boots resulted in a higher prevalence of brucellosis [43]. In all studies, multivariate logistic regression analyses were employed to estimate the strength of associations between *Brucella* infection and potential risk factors. The estimated regional risk of *B. abortus* seroprevalence displayed considerable variation, with distinct geographical patterns identified in disease prevalence among Chinese dairy herds. The prevalence of brucellosis in nothern provinces was higher than that in southern provinces (Fig. 2). Some *Brucella* isolates from southern provinces were genetically associated with strains from multiple northern provinces in China [64], suggesting cross-province *B. abortus* transmission via animal movements.

### 3.5 Public health relevance

Eight studies estimated the prevalence of antibodies against *Brucella* spp. in humans in seven provinces of China. All studies targeted occupation-associated populations, such as livestock workers or hospitalized patients. In more than half of the studies (5/8), individuals who tested positive for both RBT and SAT were classified as brucellosis-positive. Half of the studies utilized a non-random, convenience sampling method to enroll participants. The median seroprevalence of brucellosis among these investigated people was 3.7% (Range: 1.8–16.4) (Table 4).

**Table 4:** Study characteristics of seroprevalence of *Brucella* infection in Chinese humans.

Study period	Study type	Province	Tests	Source population	Population selection method	Sample size	Prevalence (%)	References
2004–2010	Cohort	Zhejiang	RBT + SAT	Occupation-associated	Convenient sampling	10,430	2.0	[32]
2005	Cross-sectional	Fujian	SAT	Occupation-associated	Convenient sampling	1321	3.2	[67]
2008–2020	Cohort	Shannxi	RBT + SAT	Occupation-associated	Convenient sampling	179,907	4.3	[62]
2010–2012	Cohort	Sichuan	RBT + SAT	Occupation-associated	Convenient sampling	450	4.4	[68]
2010–2014	Cohort	Inner Mongolia	SAT	Occupation-associated	Stratified randomized sampling	838,956	3.6	[69]
2012–2016	Cohort	Inner Mongolia	RBT + SAT	Suspected populations	Convenient sampling	1,102,304	3.8	[70]
2013	Cross-sectional	Inner Mongolia	RBT + SAT	Occupation-associated	Census sampling	13,098	1.8	[71]
2014–2021	Cohort	Shannxi	RBT + SAT	Occupation-associated	Convenient sampling	4263	1.4	[72]
2016	Cross-sectional	Jiangsu	RBT + SAT	Occupation-associated	Convenient sampling	895	16.4	[73]
2016–2020	Cohort	Fujian	SAT	Occupation-associated	Not available	4934	1.7	[74]
2019–2020	Cross-sectional	Shanxi and Xinjiang	SAT	Occupation-associated	Simple randomized sampling	2384	2.6	[75]

RBT: Rose Bengal Test; SAT: Serum Agglutination Test.

**Fig. 4:**
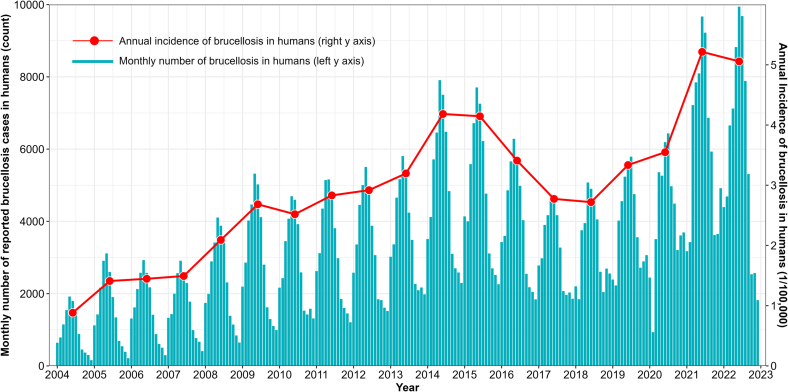
Monthly number of human brucellosis notifications in China between January 2004 and December 2022 (cyan bar: monthly number of cases by the left y-axis, red line: annual incidence of brucellosis in humans by the right y-axis, 1/100,000 people). (For interpretation of the references to colour in this figure legend, the reader is referred to the web version of this article.)

The incidence of notified human brucellosis cases displayed a typical seasonal increase with peaks in June–July (summer) and troughs in December–January (winter) each year (Fig. 4), followed by a decline during 2015–2018, and another rise through the present (Fig. 4). The historical peak occurred in 2021, with 73,645 human brucellosis cases notified, equivalent to approximately 5.22 cases per 100,000 person-years (Fig. 4). Although the average incidence was lower than that in other countries, such as Iraq and Jordan with >25 cases /100,000 person-years [4], in some regions of China (e.g. Inner Mongolia, 40.9 cases/100,000 person-years) it was comparable to severely affected countries [13]. A higher incidence of human brucellosis was found among livestock-related practitioners, males aged 35–54 and those residing in specific geographical areas (e.g. Inner Mongolia) [13,27,65]. For occupationally at-risk groups, behaviors such as consuming raw milk, assisting in calving, and handling aborted products without appropriate PPE were risk factors for *Brucella* infection [63]. In contrast, frequent disinfection of calving sites and proper disposal of aborted calves were identified as protective factors. Additionally, some meteorological and geographic factors, such as moderate altitude, grassland, medium temperatures, and reduced sunshine, were significantly associated with the incidence of human brucellosis [65,66].

## 4 Discussion

This scoping review provides a comprehensive overview of studies investigating the prevalence of antibodies against *B. abortus* and species and biovars of circulating *Brucella* spp. strains in dairy cattle and humans from literature published between 1st January 2004 and 31st December 2022. Out of the 1360 studies retrieved, only 60 were eligible for inclusion in this review. The exclusion of such a large number of studies may be due to unrelated research topics, heterogeneous quality of study reporting, and inexplicit or missing information (Fig. 1). Most of the studies were published in local journals and not possibly in English, making these results less accessible to international researchers. Therefore, this review aggregated the retrieved data that were less accessible by international colleagues to provide a more thorough understanding of *B. abortus* infection in dairy cattle and humans in China. *Brucella* spp. poses a significant global One Health threat to both bovines and humans [76]. In China, while *B. melitensis* has acquired attention and stringent control measures in sheep and goat flocks, *B. abortus* has often been neglected as a pathogen in cows and humans, particularly when compared to *B. melitensis* [77,78]. Justifying a focus on *B. abortus* becomes essential within a One Health framework of brucellosis control in China, especially considering the prevailing gaps in understanding the relative importance of different *Brucella* species in cows and humans within China. The increasing dairy population, booming milk demand, and comparative opportunities in facilitating One Health management of brucellosis underline the significant role of *B. abortus* in China. Recognizing the indispensable role of *B. abortus* in One Health management of brucellosis, this scoping review has integrated available reports to refine the current understanding of prevalence and distribution of *B. abortus* in dairy cows and humans relative to other *Brucella* species nationwide and lay the foundation for future evidence-based disease management strategies in China.

Brucellosis has been recognized as a priority disease by the Ministry of Agriculture and Rural Affairs (MARA) of China, given the notable increase in notifications among humans and reported prevalence in livestock [79]. Since 2004, the notified brucellosis cases in humans and animals exhibited key characteristics: (1) a substantial increase in incidence and prevalence; (2) seasonal fluctuations with high incidence in summer and low incidence in winter; (3) spatial expansion from northern to southern provinces [13,27,65]. The underlying causes were likely multifaceted and may be attributed to several factors, such as: (1) increased stocking density and associated higher contact frequency between animals, accelerating within-herd transmission of infectious diseases [26,65]; (2) thriving inter- and intra-provincial live animal trade and movement, which likely facilitated disease transmission between herds [13]; (3) low vaccination coverage, failing to establish sufficient herd immunity to resist *B. abortus* (re-)invasion [80]; (4) inadequate financial resources, making test-and-slaughter strategies impractical and unaffordable [80]; (5) vulnerable on-farm biosecurity measures, struggling to prevent pathogen (re-)invasion [81]; (6) limited farmer awareness about the public health and economic significance of *B. abortus*, compromising the adoption of disease control measures [82]. However, these factors may not fully explain the observed fluctuations in brucellosis notifications in humans between 2015 and 2022. Financial limitations and competing priorities, such as the current COVID-19 pandemic, may exacerbate the challenges associated with controlling and preventing brucellosis in China. The limited financial resources available to implement effective control measures, coupled with a focus on the COVID-19 response, may further hinder the prevention and control of brucellosis after January 2020.

The median reported animal-level prevalence of antibodies against *Brucella* in dairy cattle in China was 1.5%, comparable to the 1.9% reported in a previous meta-analysis [83]. However, the median reported farm-level prevalence reached 10.2%, indicating that a considerable proportion of dairy populations were affected by *Brucella* spp. Although these prevalences were lower than those reported in endemic countries such as India (animal-level: 15.1%, herd-level: 32.9%) [84] and Ethiopia (2.6% and 16.3%) [85], or other countries in the early eradication stage like New Zealand (15% and 59%) [22] and Australia [86], China still confronts a significant challenge in controlling brucellosis because of its sizable dairy population and complex conditions. Furthermore, prevalence is influenced by the incidence, average disease duration, and increased culling rate of test-positive animals. From a production standpoint, brucellosis-positive cows can cause a 30%–80% pregnancy loss, and 10%–30% milk losses [3,87], thereby having significant economic implications on the dairy industry. The magnitude of economic impacts depends on various parameters, including disease prevalence, increased abortion rate, decreased milk production, milk, meat and animal prices, labor costs, and vaccination [88]. Unfortunately, these parameters are not readily available or tailored to the Chinese unique epidemiological and socioeconomic context.

Another knowledge gap is the underreporting of farm-level prevalence of *B. abortus* infection in the dairy sector. The reasons for the underreporting are unclear, and we hypothesize that some researchers may undervalue the importance of farm-level prevalence or may be concerned that reporting a high farm-level prevalence could raise public concern. The lack of farm-level prevalence data made epidemiological information incomplete and hindered a thorough and transparent understanding of disease status at the farm level, as most control measures were designed and implemented on a farm-level basis [20]. Selective reporting could cause adverse effects and mislead policymakers and stakeholders in their decision-making. Therefore, transparent reporting of farm-level prevalence and detailed information on the investigated farms should be strengthened to acquire a robust understanding of the disease status in the population.

Prevalence estimates are often influenced by various factors such as sampling methods, target populations, testing assays, and case definitions. Small herd size has been reported to be a risk factor for *Brucella* positivity at the farm level in some studies [43,58] but not in other international studies showing increased risk with large herd size [89]. Differences in livestock management practices and levels of farm biosecurity may explain this discrepancy [80]. The age of animals sampled is essential since older animals are expected to have a longer exposure time, making them more likely to be positive to *B. abortus* [9,53,63,65]. Unfortunately, many prevalence estimate studies have not mentioned the age of animals, which may introduce biases that cannot be accounted for. Reporting as much detail as possible about the study population is of great help in interpreting results and adjusting for potential biases. Additionally, provinces with a high disease prevalence, like Inner Mongolia and Shannxi province, may be overrepresented in research reports, while the lack of brucellosis highlights the need for greater transparency in reporting true *Brucella* spp. status in these unreported regions.

Using RBT and SAT in series to judge the serological status of *Brucella* spp. exposure in dairy cattle is common in China (Table 2) [13,27]. However, this testing strategy compromises overall sensitivity while improving specificity, potentially leading to false-negative results and underestimated prevalence [90]. False-negative results may result in infected cattle being retained on the farm as *Brucella* spp. reservoirs, perpetuating disease endemicity and transmission to other susceptible animals or farms [91,92]. In cases of low prevalence, the serial testing strategy is economically justified to avoid false positives at the animal level, particularly for test-and-slaughter policies. Conversely, prioritizing the identification of positive farms may favor parallel testing to enhance the possibility of detecting positive farms at the early stage of a control program. Testing strategies should be adjusted based on the real-time prevalence of *Brucella* spp. and the control aims rather than remaining fixed. Furthermore, incorporating polymerase chain reaction (PCR) or bacterial culture techniques when serology suggests a positive result can help identify species and biovar of *Brucella* [93].

Our findings indicated that *B. abortus*, specifically *B. abortus* biovar 3, was the most commonly identified strain in Chinese dairy farms over the two decades. Although this aligns with the general consensus, the research on *Brucella spp* isolation and identification for Chinese dairy populations is still limited compared to humans as shown in Fig. 2. The isolation of *Brucella spp* in dairy cattle has only been reported in five provinces, possibly due to the lack of biosafety level 3 laboratories and trained professionals required to work with this organism in other provinces. It is crucial to update our knowledge of the species and biovars of currently circulating *Brucella* strains in unreported prevalent provinces (Fig. 2A). Molecular epidemiology is essential for enhancing our understanding of brucellosis transmission and contact patterns in endemic regions that maintain molecular databanks of circulating *Brucella* spp. strains [70]. Researchers can infer transmission timing and direction by examining the molecular features and evolutionary relationships of these isolates from different farms or regions [64,78,94]. Investigating these epidemiological links will allow for more informed inferences about the most critical transmission routes in endemic areas, enabling evidence-based biosecurity recommendations tailored to individual farms to help mitigate the risk of disease introduction.

*B. melitensis* has been identified as the predominant *Brucella* spp. strain causing human brucellosis in China (Figs. 2B & 3B), aligning with previous research findings [77]. The higher virulence and infectivity of *B. melitensis* and more potential contacts with *B. melitensis* hosts (e.g., sheep and goats) may account for this dominance [6,95]. The seasonal grazing and crop-grazing patterns of small ruminants allow farmers more contact with animals relative to intensively raised dairy cows. For the general public, foodborne infection is the most common route, with infections from the consumption of raw milk frequently reported [96]. Occasional *Brucella* vaccine leaks have also been reported in China [15,16], highlighting the need to improve laboratory biosafety management. Among ten provinces, 691 strains were isolated from humans, with most of these isolates clustered in Inner Mongolia and Shannxi Provinces (Fig. 2B). Consequently, caution should be exercised in generalizing the current findings to other provinces, as the heterogeneous distribution of *Brucella* isolates across provinces may impact the broader applicability of the results (Fig. 2).

This review identified live cattle movement, trade, and shared milk tankers as potential risks for the between-farms transmission of *Brucella* spp. and other infectious diseases [43,58,97]. Understanding these factors is essential when tailoring a risk-based control scheme in China. Even if a farm has achieved brucellosis-free status, the risk of transmission between farms remains if farms are still connected via other routes (e.g., animal movements) [43]. The imbalance of meat and milk consumption, cattle population, and animal market price differences across Chinese provinces accelerate inter- and intra-provincial movement or trade of live animals and animal products. Although studies have investigated animal movement and trade patterns between Chinese pig farms and markets regarding other infectious diseases [98,99], the impact of cattle movements on *B. abortus* transmission remains unexplored in China. Assessing the effectiveness of the current measures to prevent *B. abortus* transmission through live cattle movements should be a priority to develop more targeted and effective control measures [98,100].

In March 2022, the MARA of China launched a five-year action plan for the prevention and control of animal brucellosis (2022–2026) [79]. The plan encompasses five key principles for controlling brucellosis in dairy herds: (1) identifying and eliminating *Brucella*-infected animals with appropriate compensation to disrupt the within-herd transmission cycle, (2) mandating vaccination for brucellosis-positive dairy farms to reduce disease prevalence, (3) implementing pre-movement testing of animals and preventing live animal movements from vaccinated areas (high-risk) to non-vaccinated areas (low risk), (4) maintaining continuous surveillance to maintain brucellosis-free status in farms that have achieved freedom, and (5) disseminating disease knowledge to improve farmers' awareness towards the disease. These measures align with the findings of this review and are anticipated to mitigate the impact of *B. abortus* on dairy farms and public health. Moreover, a One Health framework for brucellosis and other zoonoses should be prioritized and coordinate multifaceted efforts from farmers, veterinary, public health, and government departments to combat the current brucellosis epidemic in China [76]. Available links between human brucellosis and animal brucellosis strains are still limited, which makes it difficult to track and eliminate the source of infection in aniamls. Only joint, scientific, evidence-based contributions should be conducive to effective and resonant control strategies. Given the available evidence, *B. abortus* is the principal causative agent of brucellosis in dairy cows and should be included as an integral part in the One Health management of brucellosis in China.

Despite the countermeasures adopted by the Chinese government over the past decades, brucellosis remains endemic in China, posing ongoing challenges for public health, veterinary authorities, and farm stakeholders. A successful disease control and prevention program requires a One Health approach with collaborative efforts from public health, veterinary departments, and stakeholders. Adequate financial resources are critical, particularly for the test-and-slaughter approach, and appropriate compensation for culled animals serves as a key motivator for stakeholders to report cases and comply with control measures. Furthermore, as mandated by government policy, transparent reporting of vaccination information and effective tracking of vaccinated animals is essential to prevent animal movements from vaccinated areas to non-vaccinated areas. The Chinese government is already promoting the use of electronic tracking systems to replace the original paper documents to record animal movements. More data in the future will allow effective monitoring of animal movements and successful establishment of epidemiological links between animals and humans. However, the limited number of veterinary professionals and the large population of China constrain the capacity of surveillance and control programs to conduct farm censuses, farm registration, vaccination records, animal movement tracking, data collection, and stakeholder communication. The heterogeneity across regions and the lack of transparent and high-quality reporting further hinder stakeholders' judgment of the priority and importance of the disease. Addressing these challenges requires joint efforts, financial resources, and professional expertise to effectively tackle the *B. abortus* epidemic in China. As new policies are implemented, the self-determination to adopt either vaccination or test-and-culling measures will depend strongly on farm decision-makers' awareness and understanding of brucellosis, and further scientific evidence is required to guide farmers in making evidence-based decisions.

## 5 Conclusion

In conclusion, this scoping review provides a comprehensive summary of the current status of *B. abortus* for dairy cattle and humans in China, highlighting the need for effective and efficient control strategies to mitigate the disease's impact on public health, animal welfare and economics. The study identified *B. abortus* biovar 3 as the strain currently prevalent in dairy cattle and still a non-negligible One Health threat to humans although *B. melitensis* biovar 3 is currently the dominant strain in human infections. The key risk factors that drove disease transmission between dairy herds include cattle movement or trade, introduction of new animals, and shared facilities with other farms. While China has the financial resources and capacity to achieve control of *B. abortus* in dairy cows within certain areas, several challenges need to be addressed, such as limited veterinary professionals, inadequate reporting, and lack of transparent vaccination information. Addressing these challenges will require a One Health approach with joint efforts, increased financial and professional resources, and a high level of disease awareness. Ultimately, successful control of *B. abortus* in dairy cattle can lead to reduced disease burden in humans, improved animal welfare, increased economic sustainability in the dairy sector, and provide comparative opportunities in facilitating One Health management of brucellosis in China.

## Ethical approval

Not applicable.

## Funding

This work was supported by the China Agriculture Research System of MOF and MARA (Grant Number: CARS-37).

## CRediT authorship contribution statement

**Emilie Vallée:** Methodology, Resources, Software, Supervision, Writing – review & editing. **Cord Heuer:** Conceptualization, Methodology, Resources, Supervision, Writing – review & editing. **Youming Wang:** Conceptualization, Methodology, Resources, Supervision. **Yu Wang:** Conceptualization, Methodology, Software, Writing - review & editing. **Aizhen Guo:** Conceptualization, Funding acquisition, Methodology, Resources, Software, Supervision, Writing – review & editing. **Zhen Zhang:** Methodology, Resources, Supervision. **Chris Compton:** Conceptualization, Funding acquisition, Methodology, Supervision, Validation, Writing – review & editing.

## Declaration of competing interest

The authors declare that they have no conflicting interests.

## Data Availability

We have shared the data in supplementary material.

## Appendix A Supplementary data

Supplementary material Image 1
