# Supplementary material for: A scoping review on the epidemiology and public significance of Brucella abortus in Chinese dairy cattle and humans
Source: One Health. 2024 Jan 26;18:100683. doi: 10.1016/j.onehlt.2024.100683 (PMC11247298; doi:10.1016/j.onehlt.2024.100683)
Supplement: Supplementary file 1 — Supplementary material [file mmc1.docx]

**Table S1** Specific literature searching strategy for four Databases (PubMed, Web of Science, Scopus, China National Knowledge Infrastructure - CNKI).

| **Database: PubMed** | **Database-tailored syntax (using Boolean operators)** |
| --- | --- |
| **Term** |  |
| **1 disease** | ((((brucellosis[Title/Abstract]) OR (Brucella abortus[Title/Abstract])) OR (Bang's disease[Title/Abstract])) OR (undulant fever[Title/Abstract])) OR (Malta fever[Title/Abstract]) |
|  | **AND** |
| **2 outcome** | ((((((((prevalence[Title/Abstract]) OR (seroprevalence[Title/Abstract])) OR (incidence[Title/Abstract])) OR (epidemiolog*[Title/Abstract])) OR (risk factors[Title/Abstract])) OR (surveillance[Title/Abstract])) OR (isolat*[Title/Abstract])) OR (molecular[Title/Abstract])) OR (identification[Title/Abstract]) |
|  | **AND** |
| **3 population** | (((((((((dairy[Title/Abstract]) OR (cow?[Title/Abstract])) OR (cattle[Title/Abstract])) OR (bovine[Title/Abstract])) OR (calf OR calves[Title/Abstract])) OR (heifer?[Title/Abstract])) OR (human?[Title/Abstract])) OR (people[Title/Abstract])) OR (patient?[Title/Abstract])) OR (person?[Title/Abstract]) |
|  | **AND** |
| **4 country** | (China[Title/Abstract]) OR (Chinese[Title/Abstract]) |
|  | **AND** |
| **5 time period** | ("2004/01/01"[Date - Publication] : "2022/05/31"[Date - Publication]) |
| **Total number of identified in PubMed** | **145** |
|  |  |
| **Database: Web of Science (WOS)** | **Database-tailored syntax** **(using Boolean operators)** |
| **Term** |  |
| **1 disease** | TS = (brucellosis OR Brucella abortus OR Bang's disease OR undulant fever OR Malta fever) |
|  | **AND** |
| **2 outcome** | TS=(prevalence OR seroprevalence OR incidence OR epidemiolog* OR risk factor? OR surveillance OR isolat* OR molecular OR identification) |
|  | **AND** |
| **3 population** | TS=(dairy OR cow? OR cattle OR bovine OR calf OR calves OR heifer? OR human? OR people OR patient? OR person?) |
|  | **AND** |
| **4 country** | TS=(China OR Chinese) |
|  | **AND** |
| **5 time period** | DOP=2004-01-01 to 2022-12-31 |
| **Total number of identified** | **143** |
|  |  |
| **Database: Scopus** | **Database-tailored syntax (using Boolean operators)** |
| **Term** |  |
| **1 disease** | brucellosis OR brucella AND abortus OR bang's disease OR undulant AND fever OR malta AND fever |
|  | **AND** |
| **2 areas** | prevalence OR seroprevalence OR incidence OR epidemiolog* OR risk factor OR surveillance OR isolat* OR molecular OR identification |
|  | **AND** |
| **3 population** | dairy OR cattle OR cow OR bovine OR human OR people OR patient OR person |
|  | **AND** |
| **4 country** | China OR Chinese |
|  | **AND** |
| **5 time period** | PUBYEAR > 2003 AND PUBYEAR < 2023 |
|  | **AND** |
| **6 publication type** | LIMIT-TO ( DOCTYPE , "ar" ) OR LIMIT-TO ( DOCTYPE , "sh" ) |
| **Total number of identified in Scopus** | **248** |
|  |  |
| **Database: CNKI** | **Advanced search (using Boolean operators)** |
| **Term** |  |
| **1 disease** | 布鲁氏菌 + 流产布鲁氏杆菌 + 布鲁氏菌病 + 布氏菌属 + 布氏菌 + 布病 |
|  | **AND** |
| **2 areas** | 流行病学 + 流行率 + 发病率 + 风险因子 + 分离 + 鉴定 + 分型 |
|  | **AND** |
| **3 species** | 奶牛场 + 牛 + 奶牛 + 犊牛 + 青年牛 + 母牛 + 人 |
|  |  |
| **4 country** | 中国 |
|  | **AND** |
| **5 time period** | 2004-01-01 to 2022-12-31 |
| **Total number of identified in CNKI** | **824** |
